# Comparative mitochondrial genomic analyses of three chemosynthetic vesicomyid clams from deep‐sea habitats

**DOI:** 10.1002/ece3.4153

**Published:** 2018-06-27

**Authors:** Helu Liu, Shanya Cai, Jun Liu, Haibin Zhang

**Affiliations:** ^1^ Institute of Deep‐sea Science and Engineering Chinese Academy of Sciences Sanya China

**Keywords:** chemosymbiotic bivalves, deep‐sea, gene arrangement, mitochondrial genome, vesicomyid clams

## Abstract

Vesicomyid clams of the subfamily Pliocardinae are among the dominant chemosymbiotic bivalves found in sulfide‐rich deep‐sea habitats. Plastic morphologies and present molecular data could not resolve taxonomic uncertainties. The complete mitochondrial (mt) genomes will provide more data for comparative studies on molecular phylogeny and systematics of this taxonomically uncertain group, and help to clarify generic classifications. In this study, we analyze the features and evolutionary dynamics of mt genomes from three *Archivesica* species (*Archivesica* sp., *Ar. gigas* and *Ar. pacifica*) pertaining to subfamily Pliocardinae. Sequence coverage is nearly complete for the three newly sequenced mt genomes, with only the control region and some tRNA genes missing. Gene content, base composition, and codon usage are highly conserved in these pliocardiin species. Comparative analysis revealed the vesicomyid have a relatively lower ratio of Ka/Ks, and all 13 protein‐coding genes (PGCs) are under strong purifying selection with a ratio of Ka/Ks far lower than one. Minimal changes in gene arrangement among vesicomyid species are due to the translocation *trnaG* in *Isorropodon fossajaponicum*. Additional tRNA genes were detected between *trnaG* and *nad2* in *Abyssogena mariana* (*trnaL3*), *Ab. phaseoliformis* (*trnaS3*), and *Phreagena okutanii* (*trnaM2*), and display high similarity to other pliocardiin sequences at the same location. Single base insertion in multiple sites of this location could result in new tRNA genes, suggesting a possible tRNA arising from nongeneic sequence. Phylogenetic analysis based on 12 PCGs (excluding *atp8*) supports the monophyly of Pliocardiinae. These nearly complete mitogenomes provide relevant data for further comparative studies on molecular phylogeny and systematics of this taxonomically uncertain group of chemosymbiotic bivalves.

## Abstract 2

We show the architecture and evolution of mt genomes in subfamily Pliocardinae that colonize hydrothermal vent or cold seep. A comparative mitogenomic analysis sheds light on a tRNA translocation event and possible de novo tRNA gene emersion.

## 1 BACKGROUND

The animal mitochondrial (mt) genome is typically a circular molecule, usually containing 37 genes: 13 protein‐coding genes (PCGs) (*cox1*–*cox3*,* cytb*,* nad1*–*nad6*,* nad4L*,* atp6,* and *atp8*) of the respiratory chain, 22 tRNA, and two rRNAs (*rrnL* and *rrnS*) (Wolstenholme, 1992). In addition, one extensive noncoding “A + T‐rich” region is usually present which is known to contain elements controlling the initiation of replication and transcription (Boore, 1999; Wolstenholme, 1992). Due to their fundamental roles in oxidative phosphorylation responsible for energy production (Saraste, 1999), mt PCGs are generally thought to evolve primarily under constant purifying selection (Oliveira, Raychoudhury, Lavrov, & Werren, 2008). During the past decade, mt genomes had been intensively investigated for the study of molecular evolution, population genetics, and inferring phylogeny (Boore, 1999; Shen et al., 2017).

Vesicomyid bivalves occur globally, mostly in sulfide‐rich marine substrates found at deep‐sea hydrothermal vents, hydrocarbon seeps, and sites of organic enrichment such as whale carcasses (Johnson, Krylova, Audzijonyte, Sahling, & Vrijenhoek, 2017; Krylova & Sahling, 2010; Peek, Gustafson, Lutz, & Vrijenhoek, 1997; Peek et al., 2000). Most members of this family housing intracellular autotrophic sulfide‐oxidizing endosymbionts that provide essentially all of their nutrients and energy supply, making them primary subjects for studying adaptive strategies for chemosynthesis‐based nutrition (Krylova & Sahling, 2010), and host/symbiont coevolution (Ozawa, Shimamura, Takaki, Takishita et al., 2017; Shimamura et al., 2017). The Vesicomyidae are divided into two subfamilies: Vesicomyinae and Pliocardiinae partially according to their gut and gill structure (Krylova & Sahling, 2010). Vesicomyinae including small‐sized bivalves are characterized by nonreduced gut and the absence of subfilamental tissue in gills, whereas all Pliocardiinae studied to date have reduced gut systems. By far, more than 100 species have been described in family Vesicomyidae distributed worldwide from sublittoral zone to the hadal depths (Krylova, Sahling, & Janssen, 2010). However, their taxonomy was still fully unresolved owing to the small gene datasets used for the phylogenetic analyses (Johnson et al., 2017). The highly compact and easily accessible mt genomes could provide informative data to define their confused genera and draw their global distribution. Complete (or nearly complete) mt genomes are known from many species of bivalves, but only five are recorded from vesicomyids: *Abyssogena mariana*,* Ab. phaseoliformis*,* Isorropodon fossajaponicum*,* Phreagena okutanii*, and “*Calyptogena*” *magnifica* (single quotes denote a dubious genus assignment) (Liu, Cai, Zhang, & Vrijenhoek, 2015; Ozawa, Shimamura, Takaki, Yokobori et al., 2017).

In this study, nearly complete mt genomes were sequenced from three pliocardiin species assigned to the genus *Archivesica*:* Archivesica* sp., *Ar. gigas*, and *Ar. pacifica* according to Johnson et al. (2017). The mt genomes were annotated and compared to other available bivalve mt genomes. In this paper, we discuss our findings in nucleotide composition, gene rearrangement patterns, and mt genome evolution at the intrafamily level.

## 2 METHODS

### 2.1 Sample and DNA extraction

Clam specimens were sampled with human occupied and remotely operated vehicles (Table 1). Specimens were initially transferred to buckets containing 100% ethanol, or preserved at −80°C until used for DNA extraction. Total genomic DNA was extracted using DNA extraction kit (Tiangen, Beijing, China) for marine animals according to manufacturer's protocols.

**Table 1:** Information of *Archivesica* specimens used in the present study

Items	*Archivesica* sp.	*Ar. gigas*	*Ar. pacifica*
GenBank accession	MF959622	MF959623	MF959624
Dive number^a^	T488	A3519	V1589
Habitat	Hydrocarbon seep	Hydrothermal vent	Hydrocarbon seep
Coordinates	36.226, −122.883	27.0138, −111.414	36.736, −122.033
Depth	3,456	2,011	635 m
Region	Monterey Bay, CA, USA	Gulf of California, Mexico	Monterey Bay, CA, USA
Locality	Shephards meander	Guaymas Basin	Mount Crushmore
Collection date	10/11/2002	01/16/2000	04/08/1999
Habitat	Cold seep	Hydrothermal vent	Cold seep
Sample processing	Stored in 95% ethanol	Stored in 95% ethanol	Stored in 95% ethanol

a Dive number: V… = ROV *Ventana*; T… = ROV *Tiburon*; A… = HOV *Alvin*.

### 2.2 Long‐PCR amplifications and sequencing

The genomic DNA was used in PCR to amplify first short fragment of *cox1*,* rrnL*,* nad5,* and *nad6*. The primers for genes *cox1* and *rrnL* were found in the literatures (Bonnaud, Boucher‐Rodoni, & Monnerot, 1994; Folmer, Black, Hoeh, Lutz, & Vrijenhoek, 1994), and degenerate primers for genes *nad6* (Forward: TGCTTTTATGTAGAAATTTTTC, Reverse: TTAAAYTTYCTHGCCAACCT) and *nad5* (Forward: TGATAWTGGGWTGAGAYGG, Reverse: GCCTTRAATAAAGC A TG) were designed based on previously reported vesicomyid bivalves mtDNA (Liu et al., 2015; Ozawa, Shimamura, Takaki, Yokobori et al., 2017). Amplification involved a touchdown PCR with the following parameters: 94°C for 3 min in the first cycle (30 s in subsequent cycles), annealing temperature starting at 56°C for 45 s and decreasing 1°C each cycle to 46°C (10 cycle in totals), and 72°C for 1.5 min, followed with 25 cycles of annealing temperature at 46°C. Positive fragments were cloned into pMD‐18 vector (Takara, Dalian, China) and sequenced, and then served as template for species‐specific long‐PCR primers designing (Appendix S1).

The technique of long‐PCR amplification was used to amplify fragments *nad6‐cox1*,* cox1‐rrnL*,* rrnL‐nad5*, and *nad5‐nad6*. The PrimeSTAR® GXL DNA Polymerase kit (Takara, Dalian, China) was used to perform the long‐PCR reactions (Jia, Guo, Zhao, & Wang, 2014). The reaction conditions were set as follows: 10 μl 5 × PrimeSTAR GXL Buffer, 4 μl dNTP Mixture (2.5 mmol/L each dNTP), primers 0.2 μmol/L each, 2 μl PrimeSTAR GXL DNA Polymerase, 4 μl template DNA, ddH_2_O up to 50 μl. Cycle conditions were set up as 35 cycles of denaturation at 98°C for 10 s, annealing at 55°C for 15 s, and extension at 68°C for 8 min. Nested primers (Appendix S1) used for long‐PCR were also used to sequence the long amplicons. However, fragments between *nad5* and *nad6* failed to be sequenced, and only partial fragments were obtained for these regions.

### 2.3 Gene annotation and bioinformatics analysis

The protein‐coding and rRNA genes are annotated by blast searches in GenBank and aligned to the orthologous mt genes of bivalves. Start codons of protein‐coding genes were set at the first start codon that did not overlap with an upstream gene, and a stop codon was not allowed to overlap with downstream genes. The boundaries of the ribosomal genes *rrnL* and *rrnS* were assumed to extend to the boundaries of flanking genes as the ends of ribosomal genes were difficult to be precisely determined by DNA sequencing alone (Boore, 2006). Identification and positional confirmation of the tRNAs was accomplished by using the software of MITFI (Juhling et al., 2012) and ARWEN (Laslett & Canback, 2008).

Codon usage and nucleotide composition statistics were estimated using DnaSP5.1 (Librado & Rozas, 2009) and Microsoft Excel 2007. The DnaSP5.1 was used to calculate nucleotide polymorphism divergence, the ratio of Ka (the number of synonymous substation per synonymous site) and Ks (the number of nonsynonymous substation per nonsynonymous site). Effective number of codons (ENC) and the codon bias index (CBI) for each PCG were also determined with DnaSP5.1. The AT‐skew and GC‐skew were calculated with formulas (A − T)/(A + T) and (G − C)/(G + C), respectively (Perna & Kocher, 1995).

### 2.4 Phylogeny

For phylogenetic analysis, we downloaded most of bivalve mt genomes used to reconstruct phylogenetic trees of subclass Heterodonta from the reference (Ozawa, Shimamura, Takaki, Yokobori et al., 2017) and two Pteriomorphia species were set as outgroup. Summarizing, we included in our dataset 32 Veneroida, four Myoida, three Lucinoida, one Pholadomyoida, and the outgroup two Ostreoida. Each PCG, with the exception of *atp8*, was separately aligned with codon‐based multiple alignments implemented in Mega7.0, and the gap and divergent regions were removed using Gblocks (version 0.91b) (Castresana, 2000) under default (stringent) settings. The *atp8* gene was not included in the analyses due to the missing of this gene in several bivalves. Even though the “missing” atp8 might be found after re‐annotation (Breton, Stewart, & Hoeh, 2010), several species still lack this gene. Alignments of individual genes were then concatenated as a combined matrix. Neighbor‐joining analysis with 1,000 bootstrap replicates was performed in Mega7.0 under default setting. For maximum‐likelihood analysis, the package jModeltest 2.1.7 (Darriba, Taboada, Doallo, & Posada, 2012) and prottest 3.4 (Darriba, Taboada, Doallo, & Posada, 2011) were used to select the best‐fit model GTR + I + G and LG + I + G + F for nucleotide dataset and amino acid dataset, respectively. Maximum‐likelihood analyses were performed in Mega7.0 (Kumar, Stecher, & Tamura, 2016) with 1,000 replicates.

## 3 RESULTS AND DISCUSSION

### 3.1 The mt genomes in deep‐sea clams

The mt genomes were sequenced by a combination of short and long‐PCR amplifications. Initially, partial fragments of four mtDNA genes (*nad6*,* cox1*,* rrnL*, and *nad5*) were amplified using degenerate primers designed according to known vesicomyid mt genomes. Then species‐specific primers based on the known fragments were redesigned to perform long‐PCR amplification. Except for regions located between *nad6* and *nad5*/*trnaL1*/*trnaW*, most parts of the mt genomes were successfully sequenced (Appendix S2). The regions that failed to be sequenced were known to contain notable base composition bias and high numbers of tandem repeats (Liu et al., 2015; Ozawa, Shimamura, Takaki, Yokobori et al., 2017) that may have disrupted PCR amplification, as has been reported in animal species (Ozawa, Shimamura, Takaki, Yokobori et al., 2017; Yuan, Zhang, Guo, Wang, & Shen, 2015). As a result, 15,650, 15,674, and 17,782 bp mtDNAs were obtained for *Archivesica* sp., *Ar. gigas* and *Ar. pacifica* with an overall 64.8%, 65.0%, and 68.6% A − T content, respectively, which are consistently biased toward being AT‐rich like other mollusks.

Typical metazoan mt genomes generally contained 37 genes including 13 protein‐coding genes (PCGs), two rRNAs, and 22 tRNAs (Wolstenholme, 1992), whereas vesicomyid clams are reported to contained 37–39 genes after re‐annotation (Liu et al., 2015; Ozawa, Shimamura, Takaki, Yokobori et al., 2017) (Appendix S3). The three genomes examined in this study contained 13 PCGs (including the *atp8* gene) and two rRNA (Appendix S2), all encoded on the same strand, as consistently reported for other bivalves (Ozawa, Shimamura, Takaki, Yokobori et al., 2017; Xu, Wu, & Yu, 2010). Twenty‐two tRNAs were detected for *Ar. pacifica*, while 20 and 21 tRNA were detected for *Archivesica* sp. and *Ar. gigas*, respectively, indicating that we have sequenced most mt genes of these three clams.

**Figure 1:**
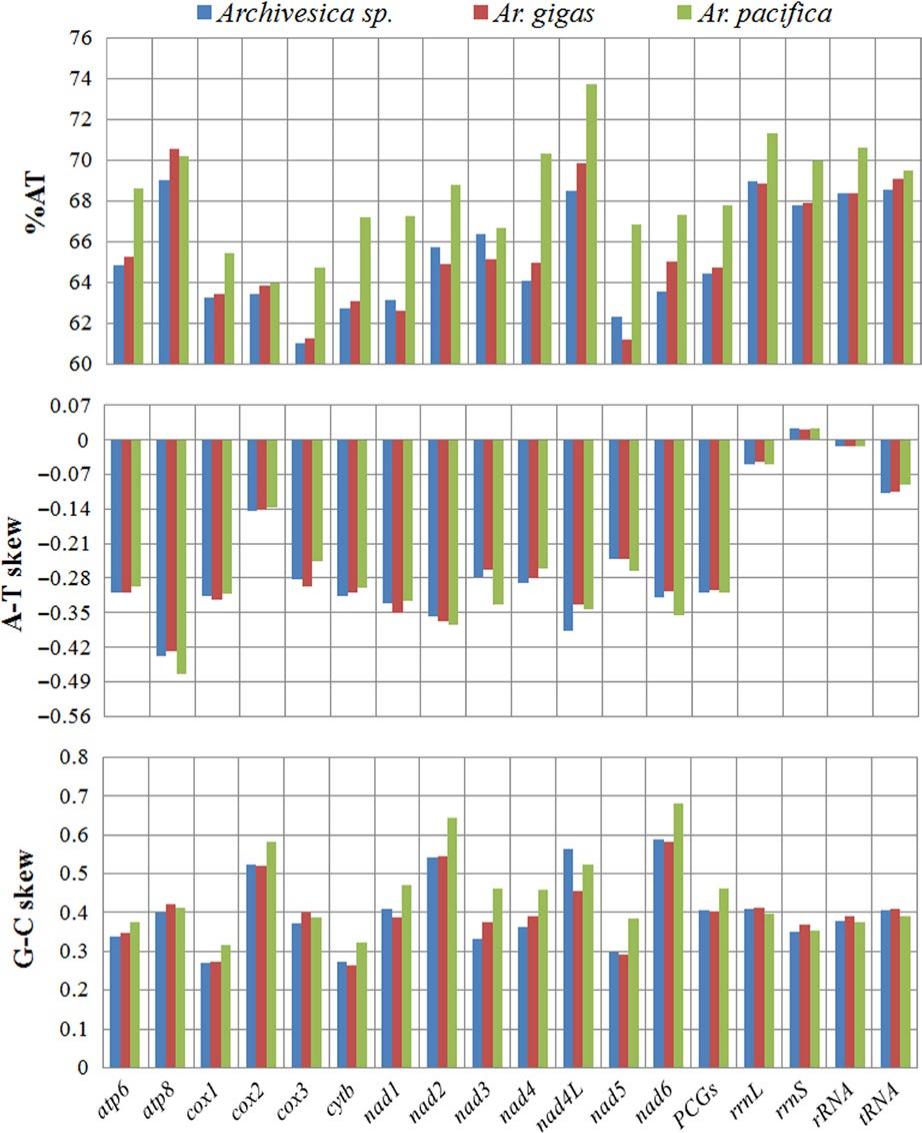
Compositional patterns of *Archivesica* sp., *Ar. gigas*, and *Ar. pacifica* mitochondrial genomes. AT content, A − T skew, and G − C skew are computed for each protein‐coding genes (PCGs) and rRNAs, and for PCGs, rRNA and tRNAs taken as a whole

Nucleotide composition and A − T/G − C proportions were computed for each single gene and for PCGs, rRNA, and tRNAs taken as a whole (Figure 1). Each analyzed gene was consistently biased toward being AT‐rich and positive GC‐skew. All PCGs were heavily negative AT‐skewed, while the tRNA genes were moderately T‐skewed, and the rRNA genes are slightly T‐ or A‐skewed. Nucleotide composition bias is also reflected in the codon usage pattern (Appendix S4, S5). Among 62 amino acid encoding codons, UUU‐F, UUA‐L, and AUU‐I were the most frequently used codons. Relative synonymous codon frequencies (RSCU) revealed that the degenerate codon usage at the third codon positions is generally biased to use more As and Ts than Gs and Cs. However, vesicomyid species used more codon GGG for amino acid Gly than other three degenerate codons (GGA, GGC, and GGU), while some bivalves used GGU or GGA as the most frequently used codon for Gly (Plazzi, Ribani, & Passamonti, 2013; Xu et al., 2010).

**Figure 2:**
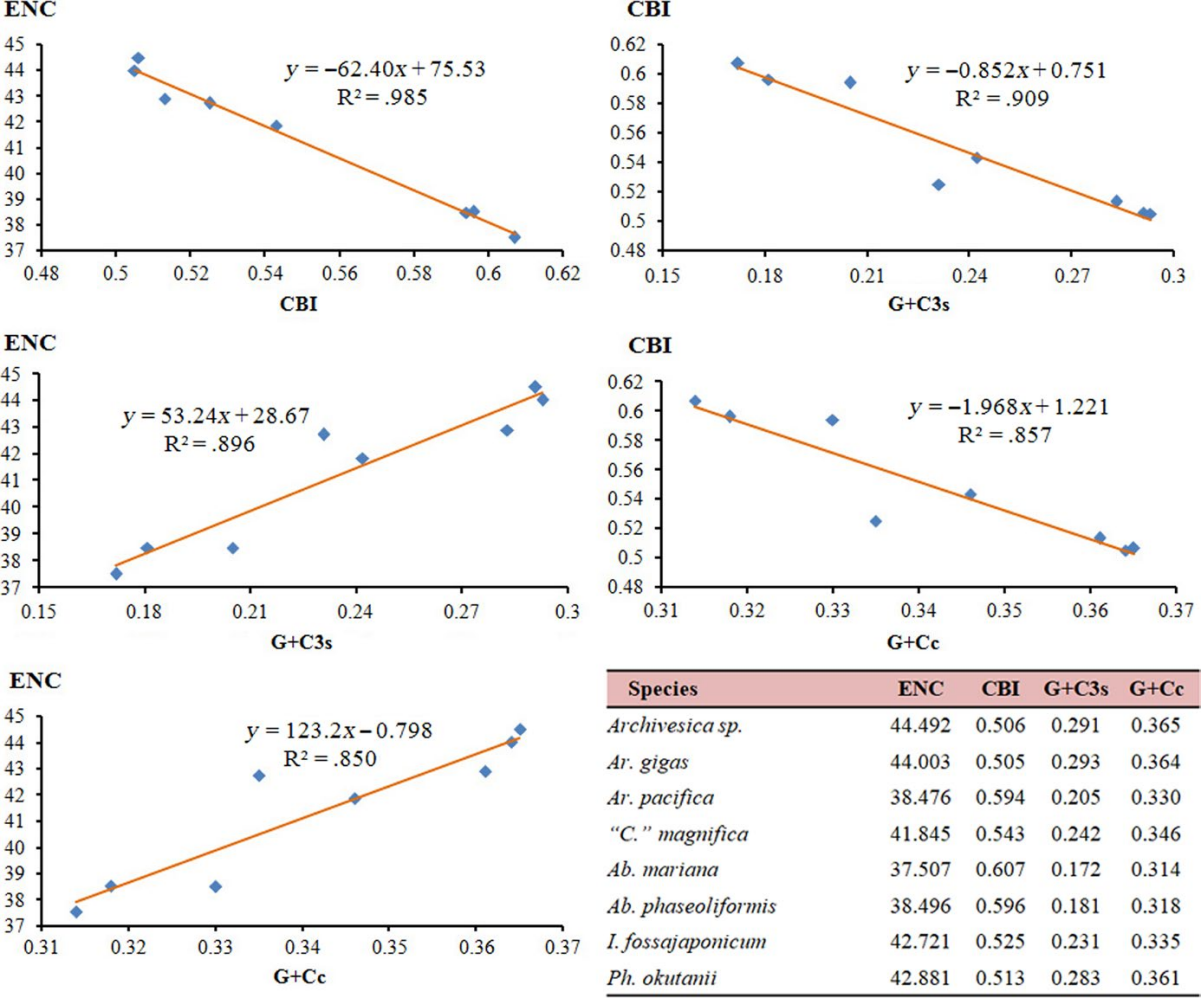
Evaluation of codon bias in the mitochondrial genomes of eight vesicomyid species. ENC, effective number of codons; CBI, codon bias index; G + Cc, G + C content of all codon positions; G + C3s, G + C content of the third codon positions

To further investigate the codon usage bias among vesicomyid species, we analyzed the correlations between ENC (effective number of codons), CBI (codon bias index), the G + C content of all codons (G + Cc), and the G + C content of the third codon position (G + C3s). We found a negative correlation between CBI and ENC (*R* ^2^ = .985), G + Cc (*R* ^2^ = .857) and G + C3s (*R* ^2^ = .909), whereas a positive correlation was found between ENC and G + Cc (*R* ^2^ = .85) and G + C3s (*R* ^2^ = .896) (Figure 2). Results were consistent with the neutral mutational theories, in which the G + C content of mt genome was reported to be the most significant factor in determining codon bias among organisms (Chen, Lee, Hottes, Shapiro, & McAdams, 2004; Plotkin & Kudla, 2011).

### 3.2 Protein‐coding genes

The vesicomyid mt genomes contained the full set of PCGs (including *atp8*) usually presented in metazoan mtDNA. We re‐annotated mt genome of “*C*.”* magnifica*, which changed the location of start and/or stop codon for *nad3* and *cox3* (Appendix S3). Except for *cox1* and *cox2*, substantial size variation for each of the PCGs was not found among the eight vesicomyid species compared in this study (Appendices [Link], [Link]). The *cox1* sequence from *Archivesica* sp., *Ar. gigas*,* Ar. pacifica*, and “*C*.”* magnifica* contained 1,806 bp, which is 150–153 bp longer than the sequence in *Ab. mariana*,* Ab. phaseoliformis*,* I. fossajaponicum*, and *Ph. okutanii*. The *cox2* sequences varied from 1,017 bp in *Ph. okutanii* to 1,395 bp in “*C*.”* magnifica*.

Most PCGs in all eight vesicomyid mt genomes started with a typical metazoan codon ATN, and ATG was the most used. Compared with other five vesicomyid clams, *Ab. mariana*,* Ab. phaseoliformis*, and *I. fossajaponicum* had no initiation codon ATA (Appendix S4). GTG appeared to be initial codon for *nad4L* and *nad5*. Most PCGs ended with the termination codon TAN (TAG, *n* = 62; TAA, *n* = 34). Only *cox3* adjacent to downstream tRNA gene *trnaF* had a truncated termination codon TA in all eight vesicomyids. The truncated stop codon was common in metazoan mt genomes and might be corrected by polyadenylation during posttranscriptional processing (Dreyer & Steiner, 2006).

### 3.3 Transfer RNA genes

We re‐annotated mt tRNAs of “*C*.”* magnifica*, and a putative *trnaS* (*trnaS1*) was identified and located between *cox3* and *trnaY*. *Ar. pacifica* and “*C*.”* magnifica* had typical 22 tRNA genes of metazoan mt genomes, whereas *Ab. mariana*,* Ab. phaseoliformis*,* I. fossajaponicum*, and *Ph. okutanii* had extra *trnaL* (anticodon: UAA), *trnaS* (anticodon: UGA) and *trnaH* genes, *trnaN* and *trnaK* genes, and *trnaM* gene, respectively, besides the typical 22 tRNAs (Ozawa, Shimamura, Takaki, Yokobori et al., 2017). The average size of all tRNAs present in the vesicomyid mt genomes ranged from 60 to 71 bp. No substantial size variation existed between tRNAs, as previously reported for bivalve homologs (Xu et al., 2010). In addition, anticodon arms and aminoacyl acceptor stems in the vesicomyid tRNA sequences and structures were highly conversed (Appendix S6). Most of the nucleotide variation with obvious indel polymorphisms was restricted to the dihydrouridine (DHU) arm and pseudouridine (TψC) loops. Although the *trnaC* and *trnaG* had the most sequence variation among all tRNAs, their secondary structures were conserved (Appendix S7).

Almost all of the tRNA genes possessed the cloverleaf secondary structure composed of four arms with conserved size, except the two *trnaS* genes which appeared to lose the DHU arm (Appendix S7). Similar structures have also been observed in many other bivalve mt genomes (Plazzi et al., 2013). Although the function of tRNAs that lack a DHU arm have not been characterized in bivalves, it have been reported that in the nematode the tRNAs lacking either the DHU or the TψC arm still retained functionality (Okimoto, Macfarlane, Clary, & Wolstenholme, 1992).

### 3.4 Ribosomal RNA genes

As in other vesicomyids, the large and small rRNA subunits (*rrnL* and *rrnS*) from the three newly cloned mt genomes were located between *cytb* and *atp8*, and between *trnaT* and *trnaM*, respectively. We re‐annotated the boundaries of *rrnL* and *rrnS* of the five previously reported vesicomyid clams to have the largest frame, but we did not allow them to overlap with adjacent genes. The length of *rrnL* varies from 1,221 bp in *Archivesica* sp. to 1,256 bp in *Ab. mariana*, whereas the largest and smallest *rrnS* genes were 932 bp in *I. fossajaponicum* and 878 bp in *Pa. okutanii*. Therefore, there was not substantial size variation between rRNAs within these vesicomyid species (1,237 ± 13 bp in *rrnL* and 888 ± 18 bp in *rrnS*).

### 3.5 Evolution of the vesicomyid mt genome

**Figure 3:**
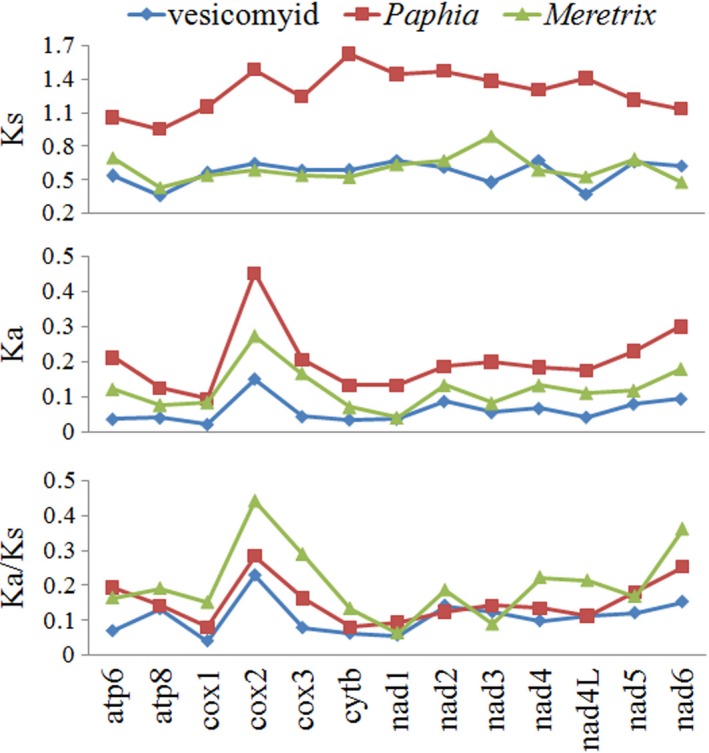
Comparison of sequence divergence for each mitochondrial gene. Vesicomyid clams including eight species. The genus *Paphia* includes *P. textile*,* P. undulata*,* P. amabilis*, and *P. euglypta*. The genus *Meretrix* includes *M. petechialis*,* M. lusoria*,* M. lamarckii*, and *M. lyrata*

The ratio of Ka (nonsynonymous substitution rate) to Ks (synonymous substitution rate) is widely accepted to measure the rate of PCGs sequence evolution: Ka/Ks > 1 means positive selection; Ka/Ks < 1 means purifying selection; and Ka/Ks not significantly different from 1 indicates neutral evolution (Yang & Bielawski, 2000; Zhang et al., 2006). We compared Ka/Ks ratios for all 13 PCGs in eight vesicomyid clam species. The average value of Ka and Ks ranged from 0.02 of *cox1* to 0.15 of *cox2* and from 0.36 of atp8 to 0.67 of *nad4*, respectively (Figure 3). The ratio of Ka/Ks of all PCGs was far lower than one (≤0.23), indicating that these genes evolved under purifying selection. For comparative purposes, we generated two similar data from four *Meretrix* species and four *Paphia* species belonging to the order Veneroida, which includes vesicomyids. These two sublittoral bivalve genera generally had a higher Ka/Ks ratio for mt PCGs, suggesting vesicomyid clams might undergo harsher purifying selection for mt PCGs. Although the vesicomyids and *Meretrix* had similar values for Ks, the vesicomyids had lower values for Ka. It appears that deep‐sea vesicomyids might be less tolerant of nonsynonymous substitution in mt PCGs than the sublittoral bivalves. The relatively low value of Ka of vesicomyid mt PCGs might result from severe sweeps of nonsynonymous mutations. Low Ka/Ks ratios were also reported for mt PCGs from the deep‐sea giant isopod *Bathynomus* sp. (Shen et al., 2017). The mt PCGs work in close association with nuclear‐encoded subunits in protein complexes involved in oxidative phosphorylation system (Burton & Barreto, 2012). Malfunctions of these PCGs would be lethal or at least severely affect fitness. Lethal mutation will be excluded, while neutral, mildly deleterious or suited mutation will be retained (Oliveira et al., 2008). Vesicomyid species usually reside in pore water that contains high concentration of toxic chemicals such as hydrogen sulfide and heavy metal, and symbioses with chemosynthetic bacteria. The toxic habitat, in combination with effect of symbionts, might serve as the force of directional selection.

### 3.6 Gene order

**Figure 4:**
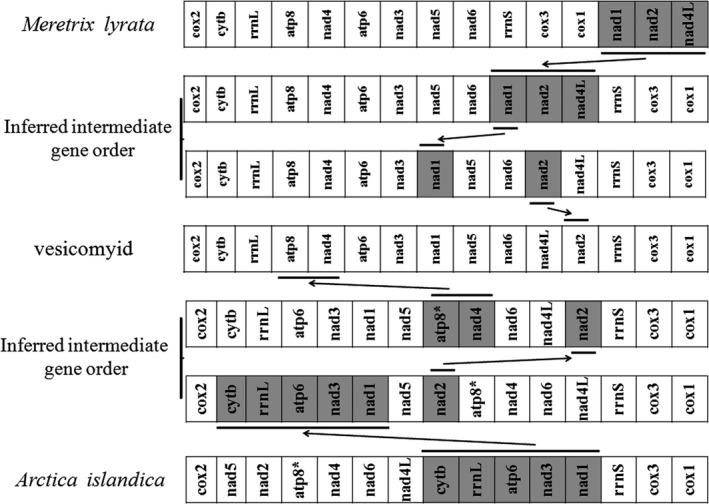
Gene rearrangements. Reconstruction of relationships among gene arrangements of vesicomyid, *Meretrix lyrata*,* Arctica islandica* is shown, after the exclusion of tRNAs. Atp8* missed in bivalve *A. islandica*

Previous work had showed that gene orders in bivalve mtDNA were highly rearranged, and tRNAs were more highly rearranged than PCGs and rRNA genes (Milbury & Gaffney, 2005). As expected, a lot of differences were found in gene arrangement between vesicomyid clams and other bivalves. After excluding tRNA genes from comparison, the eight vesicomyid species had a conversed gene order quite different from the *Solemya velum* clam (Eisen, Smith, & Cavanaugh, 1992; Plazzi et al., 2013) and *Bathymodiolus* mussel (Ozawa, Shimamura, Takaki, Yokobori et al., 2017), which were also reported to harbor chemoautotrophic symbionts in its gill tissue for their nutrition. However, they displayed somewhat similar gene order with genus *Meretrix*, sharing three gene blocks (*cox2‐cytb‐rrnL‐atp8‐nad4‐atp6‐nad3*,* rrnS‐cox3‐cox1*,* and nad5‐nad6*) (Figure 4). They also shared four gene blocks (*rrnS‐cox3‐cox1‐cox2*,* atp6‐nad3‐nad1*,* nad6‐nad4L*, and *cytb‐rrnL*) with *Arctica islandica* (Figure 4).

**Figure 5:**
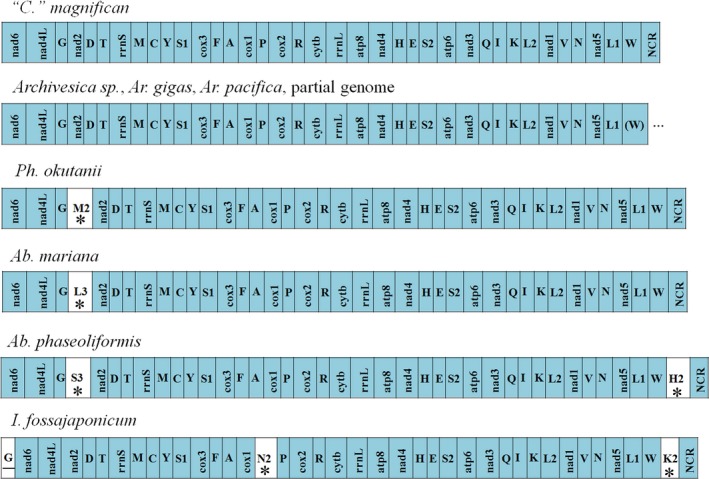
Gene order of mt genomes from eight vesicomyid clams. All species with complete or nearly complete mt genomes are listed. Noncoding region means none coding region. White colors indicate changes compared to the “*C*.” *magnifica* pattern. Asterisks indicate genes insertion and underlines refer to gene translocation event

Rearrangement of tRNA was observed within vesicomyid species. The *trnaG* located at downstream of the blocks *nad6*‐*nad4L* in most vesicomyid except for *I. fossajaponicum*, whose *trnaG* located at upstream of this block (Figure 5). By alignment, the *trnaG* in *I. fossajaponicum* was highly identical to its counterparts in other seven vesicomyid clams (Appendix S6), suggesting a tRNA translocation event. Rearrangements are random discrete events, and retro‐mutation to identical gene orders arising by chance is very unlikely (Boore & Staton, 2002). Thus, these rearrangements of tRNA in vesicomyid species would be a valuable phylogenetic marker. For example, tree topology based on the gene order was similar to that of the ML phylogeny based on the concatenated gene sequences, which was reported in bivalves (Ozawa, Shimamura, Takaki, Yokobori et al., 2017).

**Figure 6:**
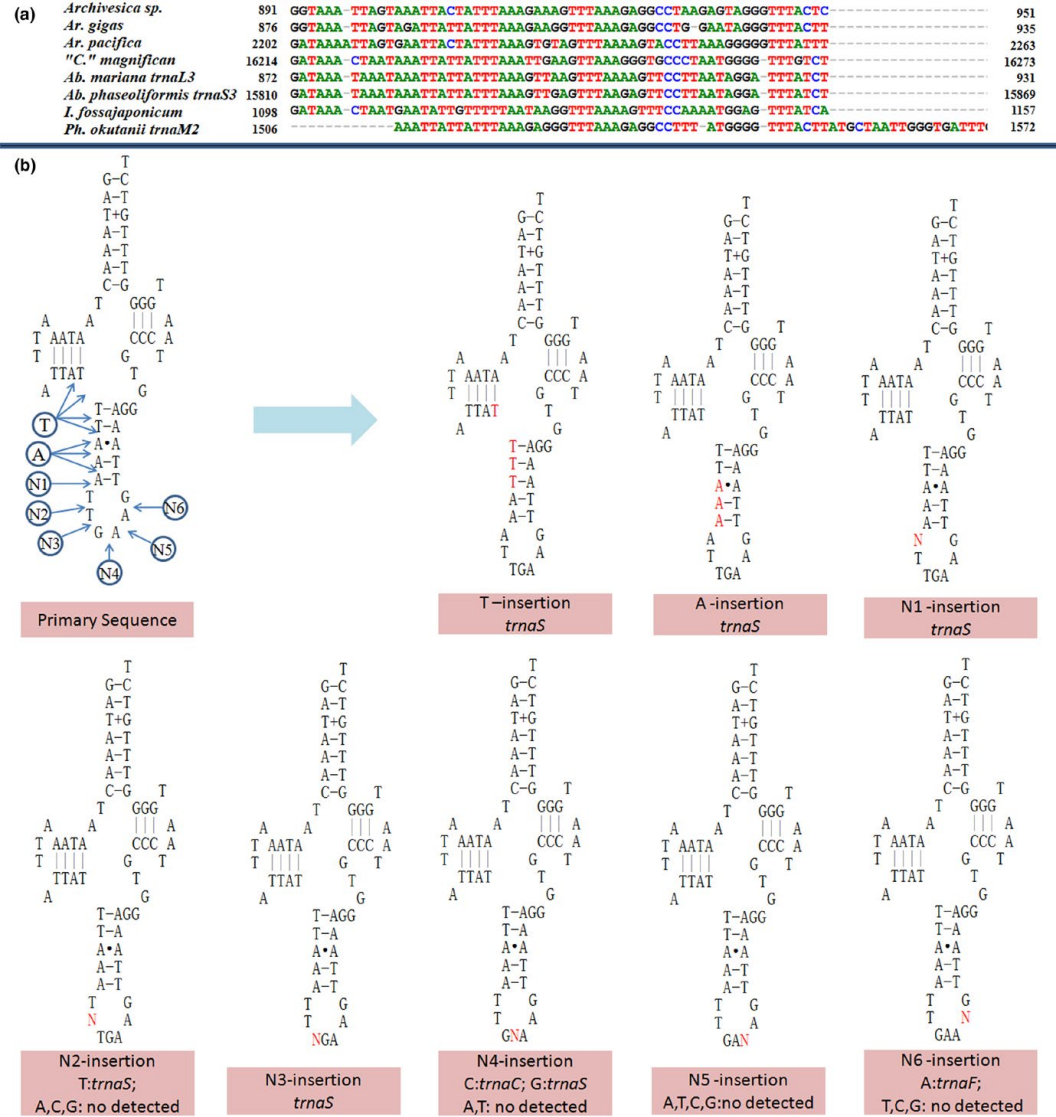
Alignment of sequence located between *trnaG* and nad2 in eight vesicomyid mt genomes (a), and tRNA detection in the putative sequence based on “*Calyptogena*” *magnifica* mt genome after base insertion (b)

When compared to “*C*.”* magnifica* mt genome, an additional tRNA genes were identified at the location between *trnaG* and *nad2* in *Ab. mariana* (*trnaL3*), *Ab. phaseoliformis* (*trnaS3*), and *Ph. okutanii* (*trnaM2*), between *cox1* and *trnP* in *I. fossajaponicum* (*trnN2*), as well as at downstream of *trnaW* in *Ab. phaseoliformis* (*trnaH2*) and *I. fossajaponicum* (*trnaK2*) (Figure 5). Additional gene copy in mt genome could be obtained by gene replication, and different gene copies would share somewhat sequence identify to each other. However, sequence analysis indicated that these additional tRNA genes showed quite a low similarity to other tRNA genes that encoded the same tRNA, instead, the additional tRNA between *trnaG* and *nad2* display high similarity to the sequences of the same location (Figure 6a). The interval between *cox1* and *trnP* in these clams except *I. fossajaponicum* is too short in length, while the intervals between *trnaW* and NCR (noncoding region) are unknown in some clams, so we do not analyze the sequence of these two intervals. The interesting point is, with a few mutations, new tRNA would be detected in the sequence between *trnaG* and *nad2*. For example, only one insertion at many sites in this region of “*C*.”* magnifica* mt genome would result in different tRNA detection (Figure 6b). Recently, comparative genomic analyses have revealed that new genes may derive from ancestral intergenic sequence (Zhao, Saelao, Jones, & Begun, 2014). The additional tRNA located between *trnaG* and *nad2* probably arise from site mutation in this region, even though more molecular evidence is needed.

### 3.7 Phylogenetic analysis

**Figure 7:**
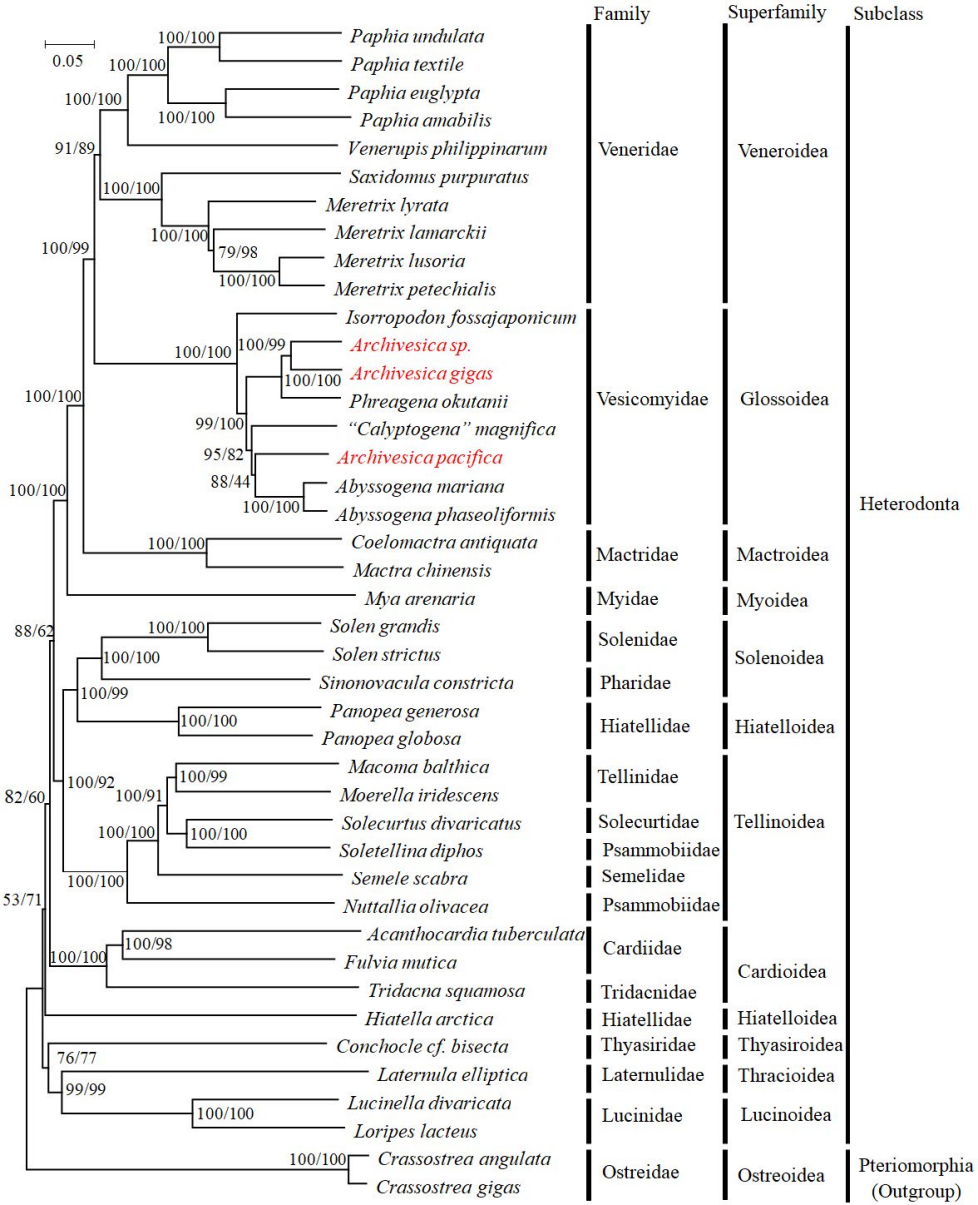
Phylogenetic relationship of Heterodonta based on the concatenated nucleotide sequences of 12 mt protein‐coding genes. Numbers on branches are bootstrap probability of Neighbor‐joining method (left) and Maximum‐likelihood method (right). Two Ostreidae species belong to the subclass Pteriomorphia are used to root the tree. Red type face indicates the species for which mt genomes are determined in the present study

Phylogenetic analyses were performed with nucleotide and amino acid sequences of 12 PCGs by NJ and ML methods. Tree topologies were not sensitive to different datasets. The nucleotide and amino acid trees are exactly the same, and support values were higher in the amino acid tree than in the nucleotide tree using the same method (Figure 7 & Appendix S8). The newly cloned vesicomyid mt genomes clustered well with the previously reported vesicomyid species with high support values. In agreement with the previous findings of phylogeny based on the concatenated 12 protein‐coding genes and nucleotide sequences of two rRNA genes (Ozawa, Shimamura, Takaki, Yokobori et al., 2017), the family Vesicomyidae grouped well with family Veneridae, then branched with family Marcridae.

## 4 CONCLUSION

In this study, we sequenced and annotated three nearly complete mt genomes belonging to family Vesicomyidae that colonize hydrothermal vents or cold seeps. Comparative analysis of eight vesicomyid mt genomes showed that gene content, codon usage, base composition, and tRNA structure were highly conserved, whereas gene arrangement displayed slight diversity. A relative low ratio of Ka/Ks exhibited by most PCGs of vesicomyid mt genomes suggested mt genomes of vesicomyid clams might undergo strong purifying selection in deep‐sea habitats. Sequence analysis showed some tRNA of vesicomyid mt genomes probably arise from ancestrally nongenic sequence.

## CONFLICT OF INTEREST

None declared.

## AUTHOR CONTRIBUTIONS

HL and SY performed all experiments and analyzed the data. HL, JL, and HZ wrote the manuscript. All authors contributed to editing and revising the manuscript. All authors read and approved the final manuscript.

## ETHICS APPROVAL AND CONSENT TO PARTICIPATE

Approval of the experimental protocol of this experiment by the Experimental Animal Ethics Committee of Hainan Province, China was not needed for this experimental approach.
