# Supplementary material for: Comparative mitochondrial genomic analyses of three chemosynthetic vesicomyid clams from deep‐sea habitats
Source: Ecol Evol. 2018 Jun 27;8(15):7261–72. doi: 10.1002/ece3.4153 (PMC6106168; doi:10.1002/ece3.4153)
Supplement: Supplementary file 1 [file ECE3-8-7261-s001.docx]

Additional file 1 Primers for long-PCR amplification

| **Species** | **Region** | **Forward primers** | **Reverse primers** |
| --- | --- | --- | --- |
| ***Archivesica sp.*** | *nad6-cox1* | Asnad6F1:GGTGGGGGCAATCGGGAAATTCTGA | Ascox1R1: CAAACCCCCCAATTATTATGGGCATTA |
|  |  | Asnad6F2:TTTTTTATTGTCCAGGTGAGCGGTA | Ascox1R2: TGCCTATCACCTAAGACTCCAGCACCA |
|  | *cox1-rrnL* | Ascox1F1: TTATTGCTATACCTGTTTTGGCTGGGA | AsrrnLR1: TCGTCAAACCTTTCATACCTTTCCCCA |
|  |  | Ascox1F2: GATTAACAATGCTATTAACTGATCGTA | AsrrnLR2: GCTATTCGCTGCAGCCGTTTAGTTA |
|  | *rrnL-nad5* | AsrrnLF2: GCAAAAGCTACCATAGGGATAACAGCG | Asnad5R1: CCTACATGCGGACACAAACACTGCTA |
|  |  | AsrrnLF1: TATTGGGGCAATAGGAAGCTAAGAAGA | Asnad5R2: GCCTCATCAACCAAATCAGGATAACGA |
|  | *nad5-nad6* | Asnad5F2: TTGAGCCAAGTGGCTAGGATGATACT | Asnad6R1:ACCTAAACCCAAAAAGACTACCTACAGA |
|  |  | Asnad5F1: GGTTGCGGGCTCAAGCTCTGCGGGAG | Asnad6R2:CAATCCTCACCATCAACAGCCCTCTA |
| ***Ar. gigas*** | *nad6-cox1* | Agnad6F1:TGCTCGGGGGGTGGGAGTAATTGGGA | Agcox1R1: AATTATTAACAACCCATGGGCAGTTA |
|  |  | Agnad6F2:AGTTTTTTTCTTATCAAGGTGAGTGG | Agcox1R2: CTACCTGGTACAAATGGCTATCCCCTA |
|  | *cox1-rrnL* | Agcox1F1: AGGCTTTATCTTTGTTACGTGTTACTA | AgrrnLR1:TCGTCAAACCTTTCATACCTTTCCCCA |
|  |  | Agcox1F2: TTGTTTGATGTGTTGCCGTGACTGGCT | AgrrnLR2: GCCGTTTAGATAAAACCACTGGGCAG |
|  | *rrnL-nad5* | AgrrnLF1:TATTGGGGCAATAGGAAGCTAAAAAGA | Agnad5R1: CTGCTAAGTCTCATGCCTCATCAACCA |
|  |  | AgrrnLF2: GCAAAAGCTACCATAGGGATAACAGCG | Agnad5R2: ATCAGGGTAACGAAGCATCACATAGA |
|  | *nad5-nad6* | Agnad5F2: TGGCCATGGGTGCTATCCAAGTTGCG | Agnad6R1:ACCTAAACCCAAAAAGACTACCTACAGA |
|  |  | Agnad5F1: ATCGACTTTGAGCCAAGTGGCGAGGA | Agnad6R2:TCTCACCCCCAATAAACCTCTCCCCA |
| ***Ar. pacifica*** | *nad6-cox1* | Apnad6F1:GGGGATGGTGGGTAAGTTTTGAAGA | Apcox1R1:CTACTTGGTACAAATGCCTATCCCCTA |
|  |  | Apnad6F2:TCCTTAATTTCTGTGACAAAGAGGGA | Apcox1R2: ACTCTAGCGCCCGGTATAGCTAACTCA |
|  | *cox1-rrnL* | Apcox1F1:GAAGCTTTATCTTTACTGCGTGTGACTA | ApS3R1:GCTTCTTTATCGTCAAACCTTTCATACCT |
|  |  | Apcox1F2:GCTATGCCTGTTTTGGCTGGGAGGTTA | ApS3R2:CTGATTTCGCTACCTTTGTACAATTATTCA |
|  | *rrnL-nad5* | ApS3F1:GGAGAAGTAAGCAAAAGTTACCATAGGGA | Apnad5R1: ACAGAACTTGAACCCGCAATCAAAACA |
|  |  | ApS3F2:AAGGTTTGCGACCTCGATGTTGGATTA | Apnad5R2: GTTCTACATGAACACACAAACACTACCA |
|  | *nad5-nad6* | Apnad5F1: TCCTGATTTGGTTGAGGAATGATGGGA | Apnad6R1:GACTTCCAACAGAAACTATTAAAGGCA |
|  |  | Apnad5F2: GGTAGTGTTTGTGTGTTCATGTAGAACTG | Apnad6R2:TAATAACCCTCCCCCTAACATAAGCG |
