# Supplementary material for: Comparative mitochondrial genomic analyses of three chemosynthetic vesicomyid clams from deep‐sea habitats
Source: Ecol Evol. 2018 Jun 27;8(15):7261–72. doi: 10.1002/ece3.4153 (PMC6106168; doi:10.1002/ece3.4153)
Supplement: Supplementary file 6 [file ECE3-8-7261-s006.docx]

*trnaA*

*
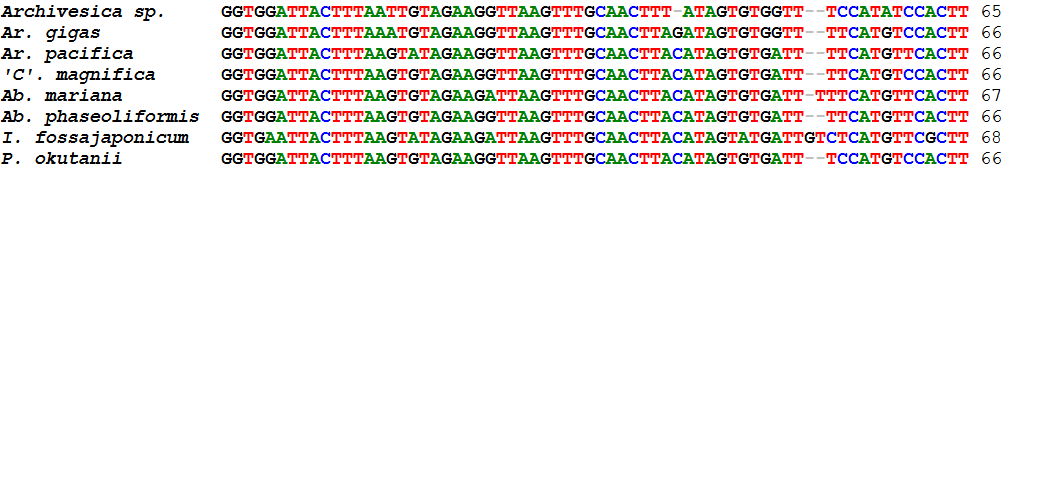
*

*----------------------------------------------------------------------------------------------------------------------*

*trnaC*

*
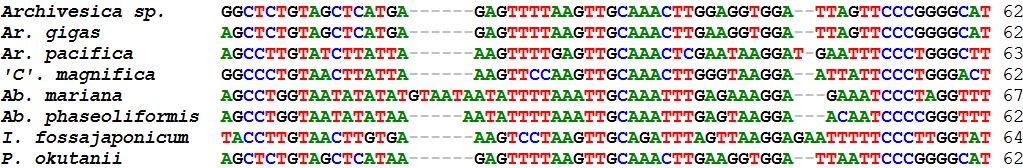
*

*----------------------------------------------------------------------------------------------------------------------*

*trnaD*

*
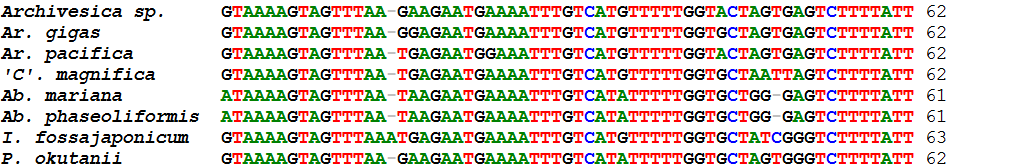
*

*----------------------------------------------------------------------------------------------------------------------*

*trnaE*

*
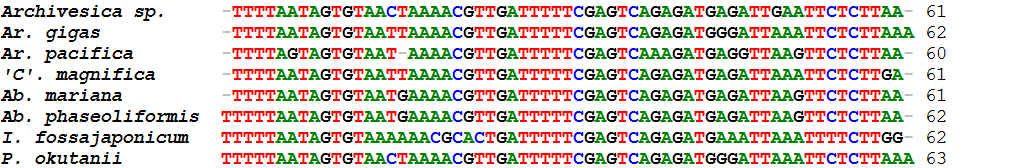
*

*----------------------------------------------------------------------------------------------------------------------*

*trnaF*

*
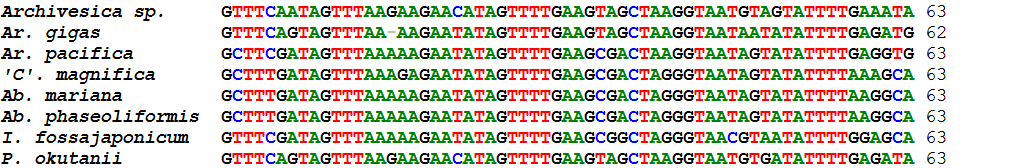
*

*----------------------------------------------------------------------------------------------------------------------*

*trnaG*

*
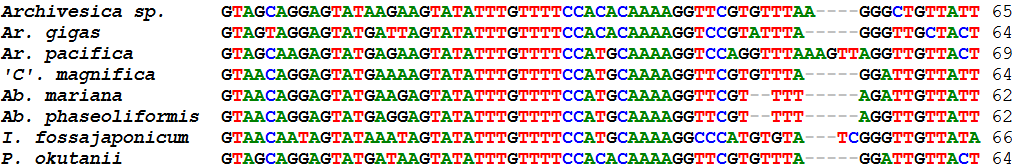
*

*----------------------------------------------------------------------------------------------------------------------*

*trnaH*

*
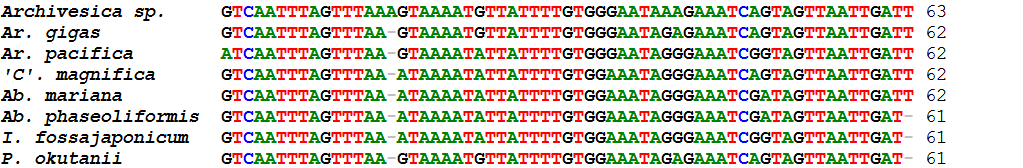
*

*----------------------------------------------------------------------------------------------------------------------*

*trnaI*

*
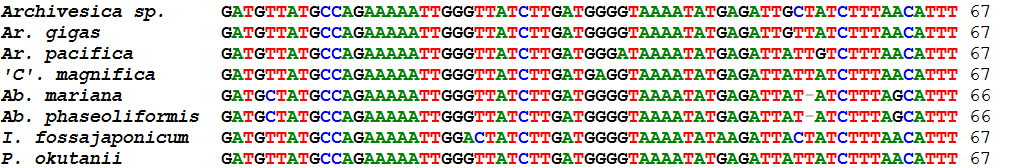
*

*----------------------------------------------------------------------------------------------------------------------*

*trnaK1*

*
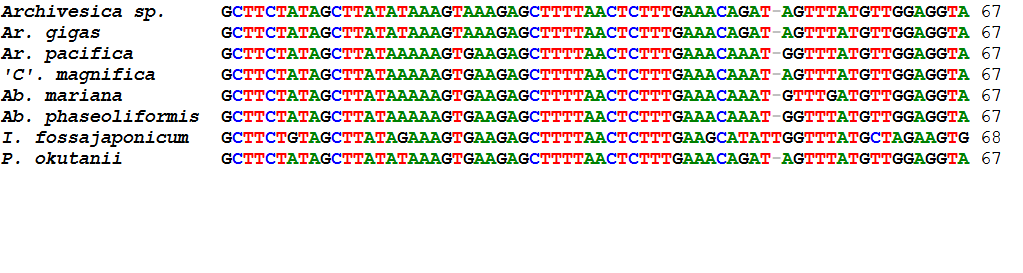
*

*----------------------------------------------------------------------------------------------------------------------*

*trnaL1*

*
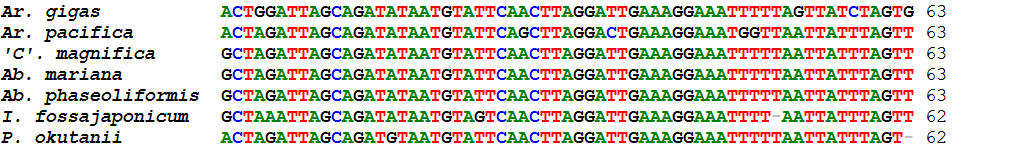
*

*----------------------------------------------------------------------------------------------------------------------*

*trnaL2*

*
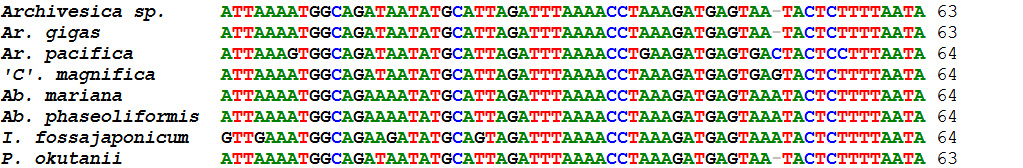
*

*----------------------------------------------------------------------------------------------------------------------*

*trnaM1*

*
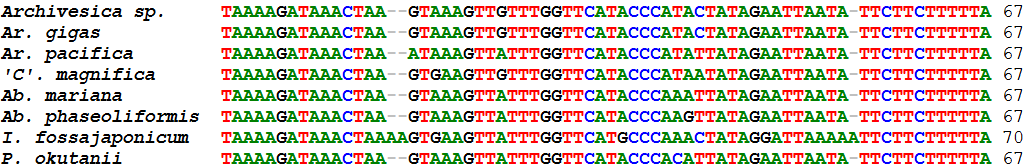
*

*----------------------------------------------------------------------------------------------------------------------*

*trnaN*

*
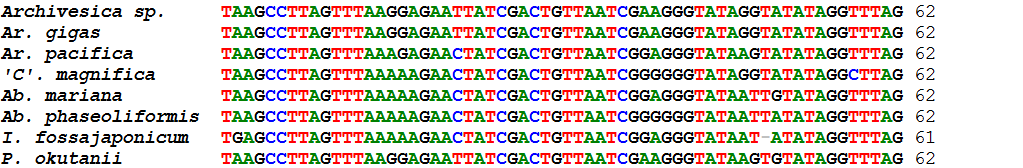
*

*----------------------------------------------------------------------------------------------------------------------*

*trnaP*

*
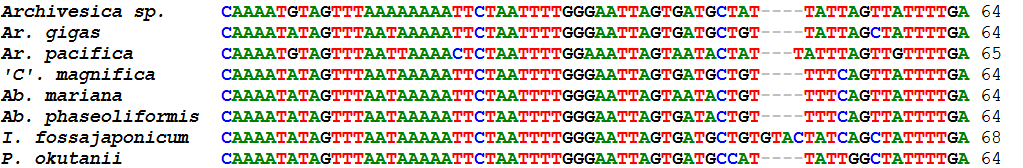
*

*----------------------------------------------------------------------------------------------------------------------*

*trnaQ*

*
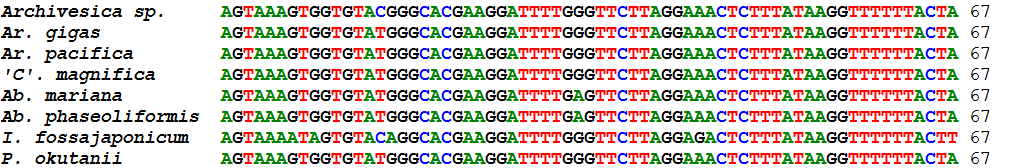
*

*----------------------------------------------------------------------------------------------------------------------*

*trnaR*

*
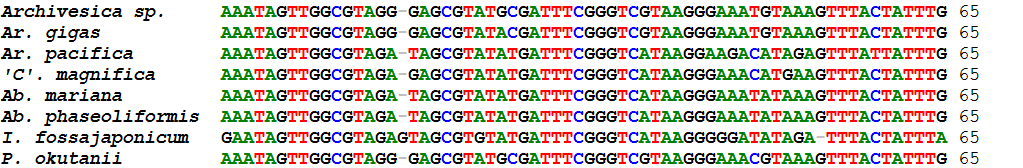
*

*----------------------------------------------------------------------------------------------------------------------*

*trnaS1*

*
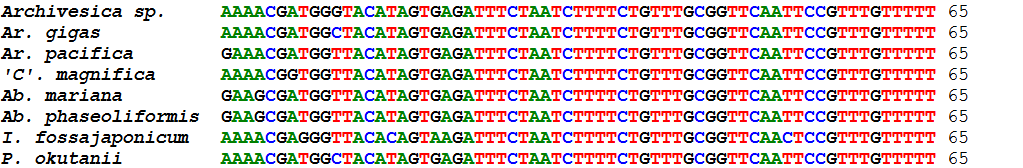
*

*----------------------------------------------------------------------------------------------------------------------*

*trnaS2*

*
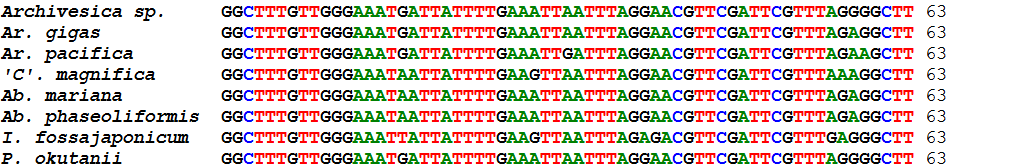
*

*trnaT*

*
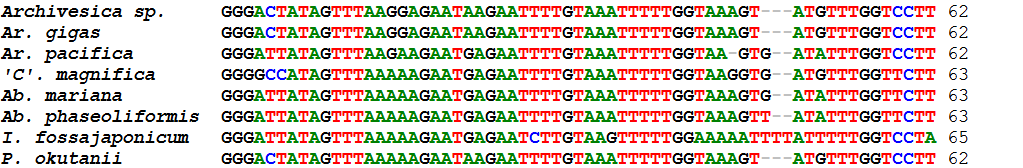
*

*----------------------------------------------------------------------------------------------------------------------*

*trnaV*

*
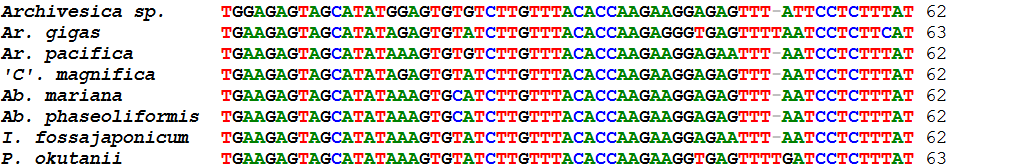
*

*----------------------------------------------------------------------------------------------------------------------*

*trnaW*

*
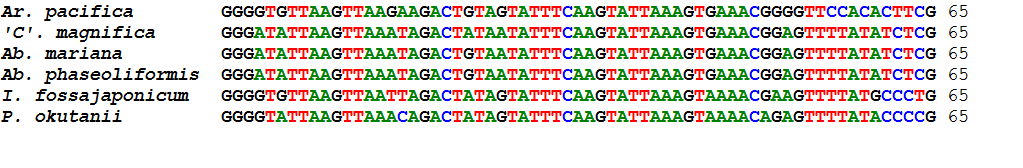
*

*----------------------------------------------------------------------------------------------------------------------*

*trnaY*

*
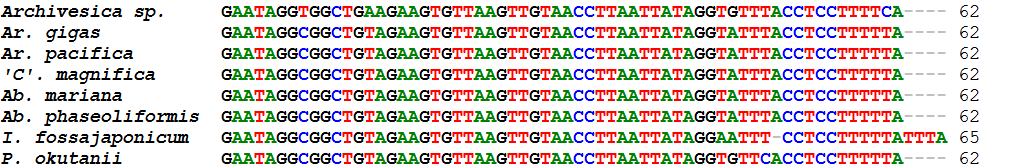
*
