# Supplementary material for: Comparative mitochondrial genomic analyses of three chemosynthetic vesicomyid clams from deep‐sea habitats
Source: Ecol Evol. 2018 Jun 27;8(15):7261–72. doi: 10.1002/ece3.4153 (PMC6106168; doi:10.1002/ece3.4153)
Supplement: Supplementary file 7 [file ECE3-8-7261-s007.docx]

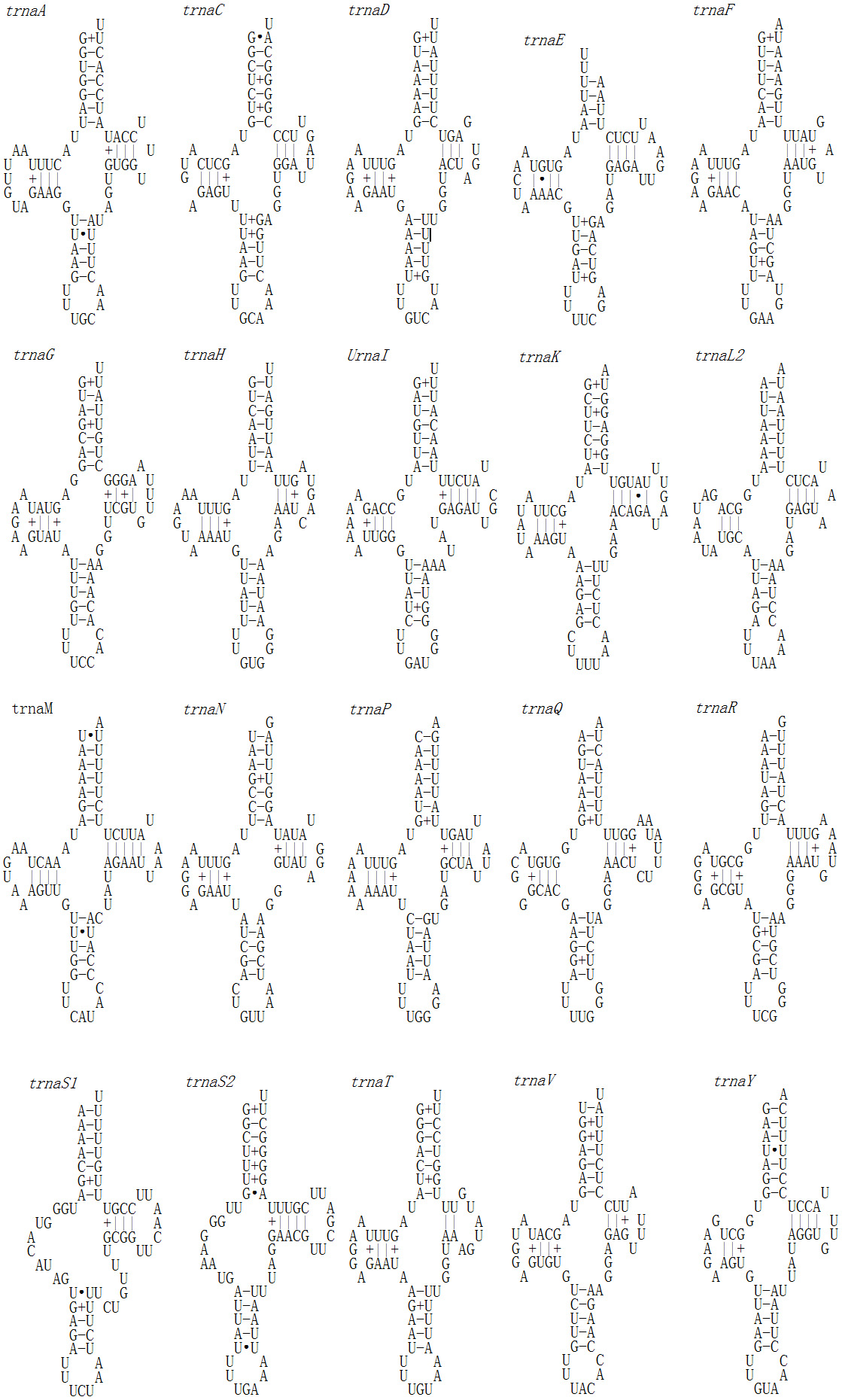


Figure 1 Proposed secondary structure of tRNA of *Archivesica sp.*. The "-" and "+" indicate Watson–Crick base pairing and a GU base pair, respectively. The "•" indicates a UU, UA or GA base pair


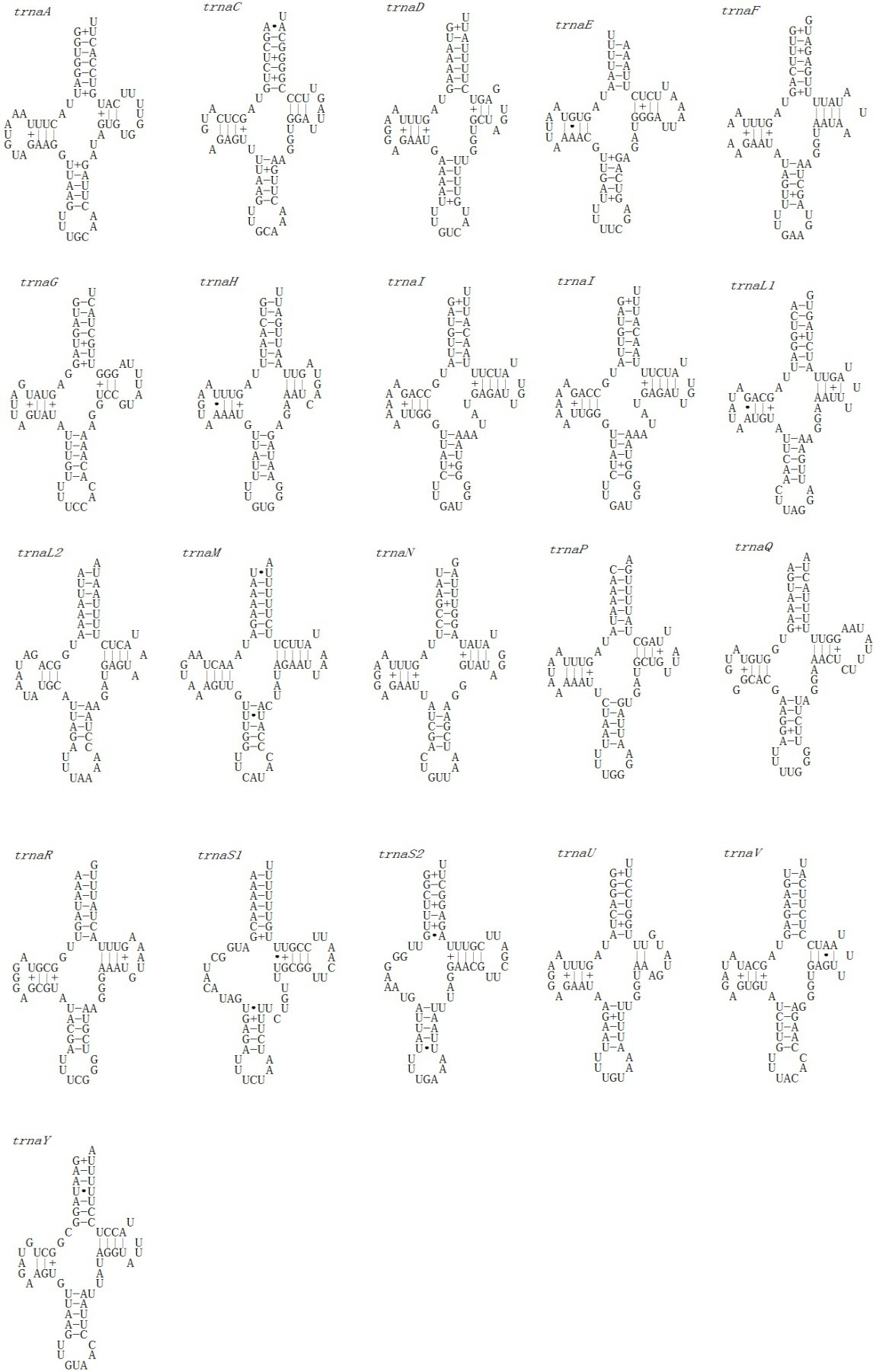


Figure 2 Proposed secondary structure of tRNA of *Ar. gigas*. The "-" and "+" indicate Watson–Crick base pairing and a GU base pair, respectively. The "•" indicates a UU, UA or GA base pair


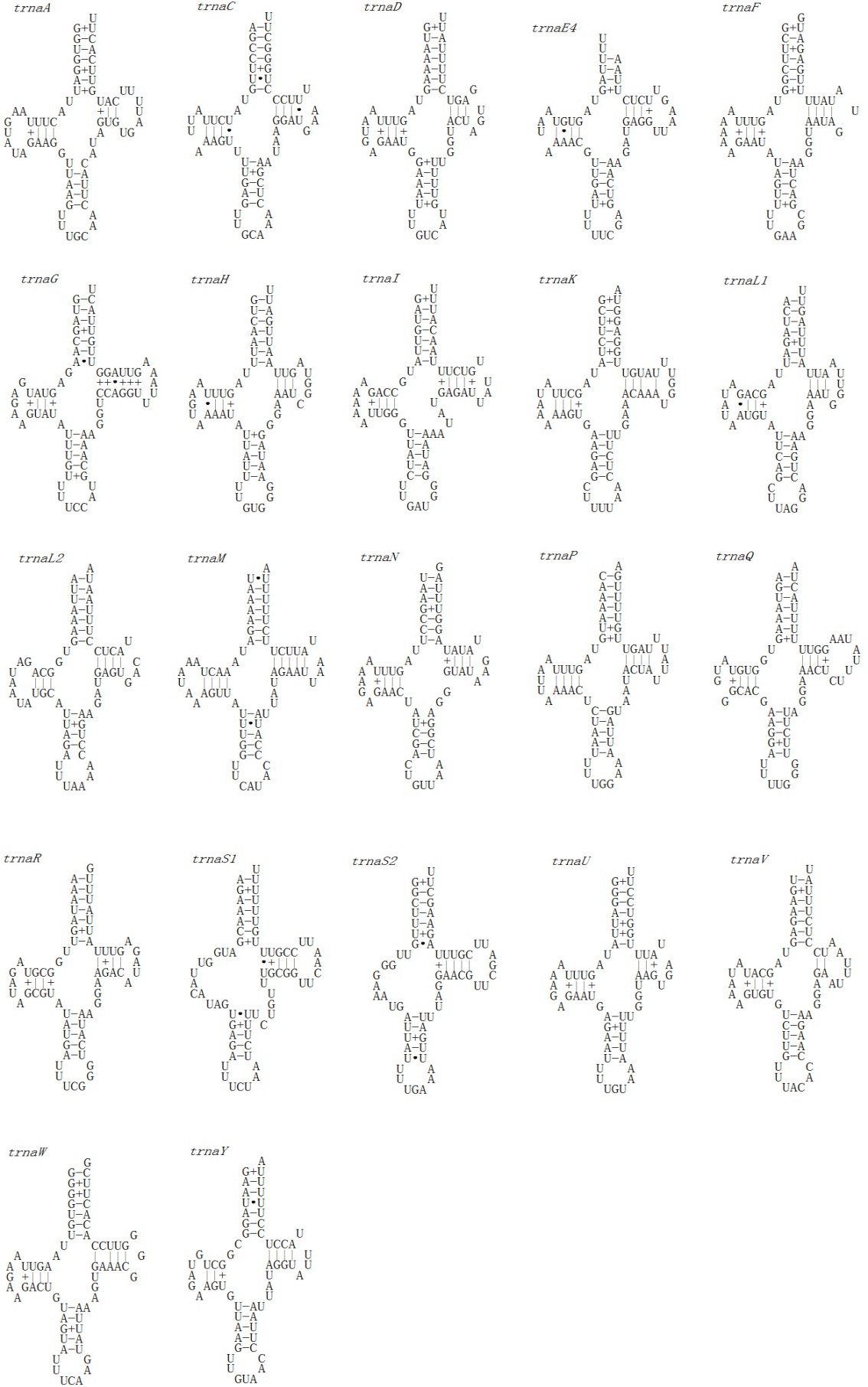


Figure 3 Proposed secondary structure of tRNA of *Ar. pacifica*. The "-" and "+" indicate Watson–Crick base pairing and a GU base pair, respectively. The "•" indicates a UU, UA or GA base pair
