# Supplementary figures and images for: Comparative mitochondrial genomic analyses of three chemosynthetic vesicomyid clams from deep‐sea habitats
Source: Ecol Evol. 2018 Jun 27;8(15):7261–72. doi: 10.1002/ece3.4153 (PMC6106168; doi:10.1002/ece3.4153)

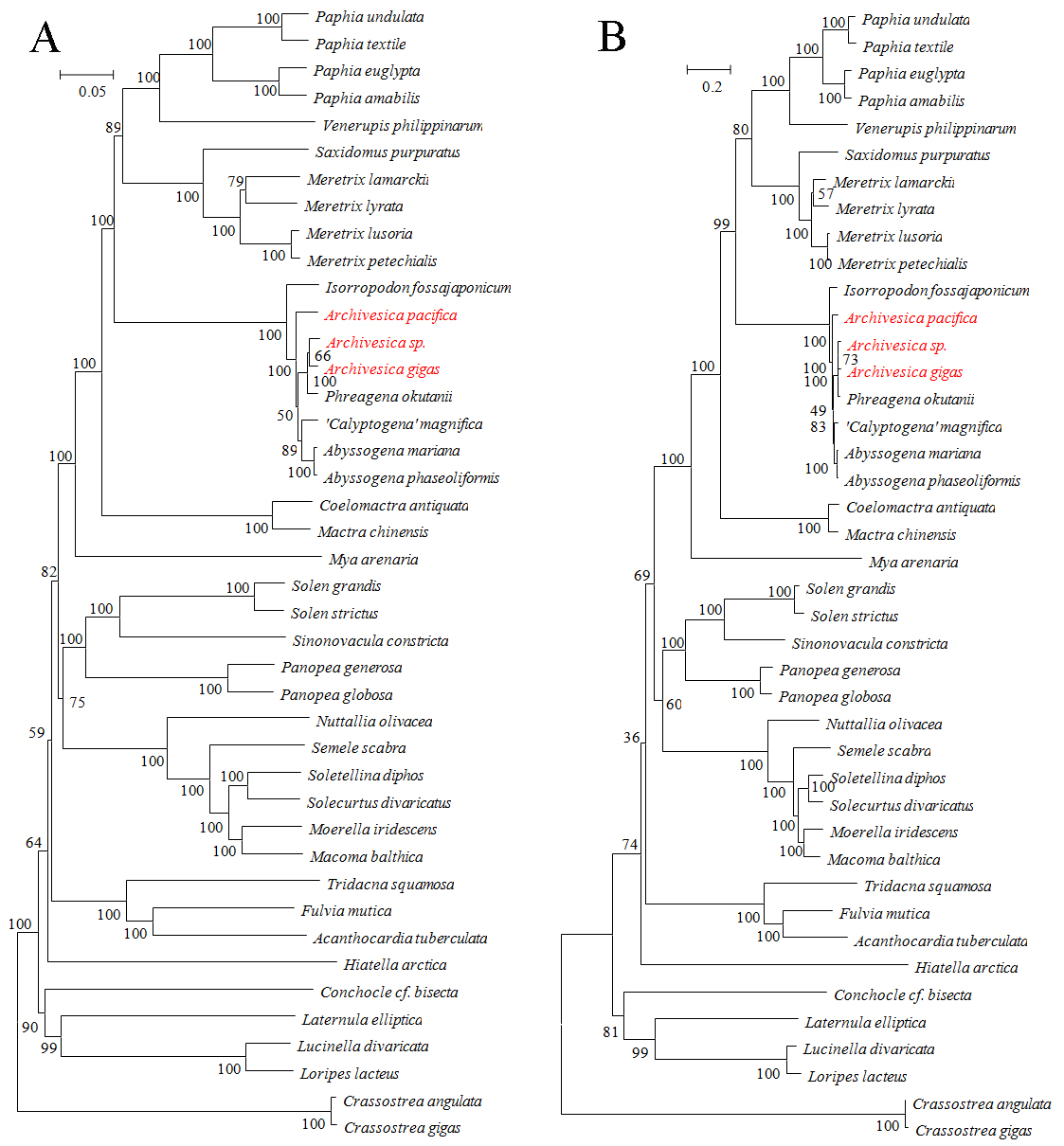

Supplement: Supplementary file 8 [file ECE3-8-7261-s008.jpg]
