# Supplementary material for: Comparative mitochondrial genomic analyses of three chemosynthetic vesicomyid clams from deep‐sea habitats
Source: Ecol Evol. 2018 Jun 27;8(15):7261–72. doi: 10.1002/ece3.4153 (PMC6106168; doi:10.1002/ece3.4153)
Supplement: Supplementary file 9 [file ECE3-8-7261-s009.docx]

List of bivalve species and their mitochondrial genomes

| Species | Accession No. | Subclass | Order | Superfamily | Family | Genome length (bp) |
| --- | --- | --- | --- | --- | --- | --- |
| *Paphia textile* | NC_016890 | Heterodonta; | Veneroida; | Veneroidea; | Veneridae | 18561 |
| *Paphia undulata* | NC_016891 | Heterodonta; | Veneroida; | Veneroidea; | Veneridae | 18154 |
| *Paphia amabilis* | NC_016889 | Heterodonta; | Veneroida; | Veneroidea; | Veneridae | 19629 |
| *Paphia euglypta* | NC_014579 | Heterodonta; | Veneroida; | Veneroidea; | Veneridae | 18643 |
| *Meretrix petechialis* | NC_012767 | Heterodonta; | Veneroida; | Veneroidea; | Veneridae | 19567 |
| *Meretrix lusoria* | NC_014809 | Heterodonta; | Veneroida; | Veneroidea; | Veneridae | 20268 |
| *Meretrix lamarckii* | NC_016174 | Heterodonta; | Veneroida; | Veneroidea; | Veneridae | 21209 |
| *Meretrix lyrata* | NC_022924 | Heterodonta; | Veneroida; | Veneroidea; | Veneridae | 21625 |
| *Venerupis philippinarum* | NC_003354 | Heterodonta; | Veneroida; | Veneroidea; | Veneridae | 22676 |
| *Saxidomus purpuratus* | NC_026728 | Heterodonta; | Veneroida; | Veneroidea; | Veneridae | 19637 |
| *Abyssogena mariana* | LC126311 | Heterodonta; | Veneroida; | Glossoidea; | Vesicomyidae | 20000** |
| *Abyssogena phaseoliformis* | AP014557 | Heterodonta; | Veneroida; | Glossoidea; | Vesicomyidae | 19424 |
| *Isorropodon fossajaponicum* | AP014550 | Heterodonta; | Veneroida; | Glossoidea; | Vesicomyidae | 23300** |
| *Phreagena okutanii* | AP014555 | Heterodonta; | Veneroida; | Glossoidea; | Vesicomyidae | 19800** |
| *Calyptogena magnifica* | KR862368 | Heterodonta; | Veneroida; | Glossoidea; | Vesicomyidae | 19738 |
| *Archivesica sp. digonalis* | MF959622 | Heterodonta; | Veneroida; | Glossoidea; | Vesicomyidae | 15650 |
| *Archivesica gigas* | MF959623 | Heterodonta; | Veneroida; | Glossoidea; | Vesicomyidae | 15674 |
| *Archivesica pacifica* | MF959624 | Heterodonta; | Veneroida; | Glossoidea; | Vesicomyidae | 17782 |
| *Coelomactra antiquata* | JN692486 | Heterodonta; | Veneroida; | Mactroidea; | Mactridae | 17384 |
| *Mactra chinensis* | NC_025510 | Heterodonta; | Veneroida; | Mactroidea; | Mactridae | 17285 |
| *Mya arenaria* | NC_024738 | Heterodonta; | Myoida; | Myoidea; | Myidae | 17947 |
| *Solen grandis* | NC_016665 | Heterodonta; | Veneroida; | Solenoidea; | Solenidae | 16784 |
| *Solen strictus* | NC_017616 | Heterodonta; | Veneroida; | Solenoidea; | Solenidae | 16535 |
| *Sinonovacula constricta* | NC_011075 | Heterodonta; | Veneroida; | Solenoidea; | Pharidae | 17225 |
| *Panopea generosa* | NC_025635 | Heterodonta; | Myoida; | Hiatelloidea; | Hiatellidae | 15585 |
| *Panopea globosa* | NC_025636 | Heterodonta; | Myoida; | Hiatelloidea; | Hiatellidae | 15469 |
| *Macoma balthica* | KM373205 | Heterodonta; | Veneroida; | Tellinoidea; | Tellinidae | 15594** |
| *Moerella iridescens* | NC_018371 | Heterodonta; | Veneroida; | Tellinoidea; | Tellinidae | 16799 |
| *Soletellina diphos* | NC_018372 | Heterodonta; | Veneroida; | Tellinoidea; | Psammobiidae | 16352 |
| *Solecurtus divaricatus* | NC_018376 | Heterodonta; | Veneroida; | Tellinoidea; | Solecurtidae | 16749 |
| *Nuttallia olivacea* | NC_018373 | Heterodonta; | Veneroida; | Tellinoidea; | Psammobiidae | 18182 |
| *Semele scabra* | NC_018374 | Heterodonta; | Veneroida; | Tellinoidea; | Semelidae | 17117 |
| *Acanthocardia tuberculata* | NC_008452 | Heterodonta; | Veneroida; | Cardioidea; | Cardiidae | 16104 |
| *Fulvia mutica* | AB809077 | Heterodonta; | Veneroida; | Cardioidea; | Cardiidae | 19110 |
| *Tridacna squamosa* | KP205428 | Heterodonta; | Veneroida; | Cardioidea; | Tridacnidae | 20930 |
| *Hiatella arctica* | NC_008451 | Heterodonta; | Myoida; | Hiatelloidea; | Hiatellidae | 18244 |
| *Conchocle cf. bisecta* | LC126312 | Heterodonta; | Lucinoida; | Thyasiroidea; | Thyasiridae | 17188 |
| *Lucinella divaricata* | NC_013275 | Heterodonta; | Lucinoida; | Lucinoidea; | Lucinidae | 18940 |
| *Laternula elliptica* | NC_022846 | Heterodonta; | Pholadomyoida; | Thracioidea; | Laternulidae | 14622 |
| *Loripes lacteus* | NC_013271 | Heterodonta; | Lucinoida; | Lucinoidea; | Lucinidae | 17321 |
| *Crassostrea angulata* | NC_012648 | Pteriomorphia; | Ostreoida; | Ostreoidea; | Ostreidae | 18225 |
| *Crassostrea gigas* | NC_001276 | Pteriomorphia; | Ostreoida; | Ostreoidea; | Ostreidae | 18224 |

** partial genome
